# Density of CD4(+) and CD8(+) T lymphocytes in biopsy samples can be a predictor of pathological response to chemoradiotherapy (CRT) for rectal cancer

**DOI:** 10.1186/1748-717X-6-49

**Published:** 2011-05-16

**Authors:** Koji Yasuda, Takako Nirei, Eiji Sunami, Hirokazu Nagawa, Joji Kitayama

**Affiliations:** 1 Department of Surgery, Division of Surgical Oncology, University of Tokyo, Japan

## Abstract

**Background:**

Although preoperative radiotherapy (RT) is widely used as the initial treatment for locally advanced rectal cancer (RC) in the neoadjuvant setting, factors determining clinical response have not been adequately defined. Radiosensitivity has recently been shown to be greatly affected by immune function of the host.

**Methods:**

In 48 cases of advanced RC, we retrospectively examined the density of tumor infiltrating CD4(+) and CD8(+) T cells using immunohistochemical staining of biopsy samples before CRT, and examined the correlation with tumor response.

**Results:**

The numbers of both CD4(+) and CD8(+) tumor-infiltrating lymphocytes (TIL) in pre-CRT biopsy samples were strongly correlated with tumor reduction ratio evaluated by barium enema. Moreover, the densities of CD4(+) and CD8(+) TIL were significantly associated with histological grade after CRT. The density of CD8(+) TIL was an independent prognostic factor for achieving complete response after CRT.

**Conclusions:**

In RC patients, T lymphocyte-mediated immune reactions play an important role in tumor response to CRT, and the quantitative measurement of TIL in biopsy samples before CRT can be used as a predictor of the clinical effectiveness of CRT for advanced RC.

## Introduction

Previous studies have demonstrated that preoperative radiotherapy (RT) can produce down-staging in advanced rectal cancer (RC), resulting in longer survival, a reduced rate of postoperative local recurrence. Recently, adding chemotherapy to RT (CRT) has achieved even more favorable results [1-3]. Thus, preoperative RT in the neoadjuvant setting is currently recognized as the standard treatment for locally advanced RC. However, in unresponsive cases, it may have disadvantages such as delaying surgery or immune suppression. Although many clinical factors [4,5], radiologic findings [6,7] and molecular markers [7-10] have been suggested to be related to therapeutic response, the clinical usefulness of these markers remains controversial, and thus identifying factors predicting the efficacy of neoadjuvant CRT is essential for decision-making in the management of patients with RC.

Recent studies have demonstrated that radiosensitivity is greatly affected by immune function of the host [11,12]. In fact, we recently showed that the circulating lymphocyte count is an important parameter determining the clinical outcome of RC patients who undergo CRT [13]. This fact inspired us to evaluate the relation between the response and the characteristics of tumor-infiltrating lymphocytes (TIL) in rectal tumors. In this study, we used immunohistochemical staining and examined the distribution and cell density of CD4(+) and CD8(+) TIL in biopsy samples before the start of CRT.

## Materials and methods

### Patients

Forty eight consecutive patients with rectal adenocarcinoma who received preoperative chemoradiotherapy (CRT) between November 2005 and August 2009 and following surgery in Tokyo University Hospital were included in this study. All the patients received a total dose of 50.4Gy radiation and concomitant 5-Fu-based chemotherapy. Among the 48 cases, 46 underwent total mesorectal excision at 6~8 weeks after the end of CRT in the Department of Surgical Oncology. In 6 cases, no tumor cells were detected at either the primary site or in regional lymph nodes on pathological examination, confirming pathological complete response (pCR). Two other patients showed a clinical CR (cCR) after CRT, with no detectable cancer cells in multiple biopsy specimens, and were thus followed without surgery and showed no evidence of recurrence for more than 16 months. In all cases, a barium enema (BE) was performed before and after CRT, the longitudinal dimension of the rectal tumor was measured on BE images before (A) and after (B) CRT, and the reduction rate was calculated as (A-B)/A.

Biopsy samples were obtained at 3-17 days before the start of CRT, and serial-step sections of the biopsy samples were cut with 3 μm width, fixed in 10% formalin solution, then embedded in paraffin, stained with hematoxylin-eosin, and the grade of tumor response was evaluated by pathologists according to the definitions in the Japanese Classification of Colorectal Carcinoma [14]: Grade 0, no remarkable changes; Grade 1, swelling of cells, enlarged vesicles, pyknosis of nuclei and vacuolated cytoplasm (< 2/3 of tumor cells); Grade 2, cell nests consisting of markedly damaged cells, often exhibiting a moth-eaten appearance and simplified granular structures in more than 2/3 tumor cells; and Grade 3, extensive degenerative changes and replaced by granulomatous or fibrous tissue. This study was performed with the approval by the Ethics Committee of the University of Tokyo, and written informed consent was obtained from the patient for publication of this case report and accompanying images. A copy of the written consent is available for review by the Editor-in-Chief of this journal.

### Immunohistochemical study of human samples

The distribution and density of CD4(+) and CD8(+) lymphocytes in biopsy samples of primary rectal tumor were evaluated by immunohistochemical staining using affinity purified mouse monoclonal antibodies against CD4 (1F6, mIgG1) and CD8 (4B11, mIgG1) (Novocastra, CA). The specificities of these mAbs in immunohistochemistry on paraffin embedded samples were confirmed with human tonsil tissue sections (data not shown). Sections (3 μm thick) at the center of the the biopsy specimens were deparaffinized in xylene, hydrated through a graded series of ethanol, and heated in a microwave oven for two 7-minute cycles (500 watts). After rinsing in phosphate buffered saline (PBS), endogenous peroxidase activity was inhibited by incubation with 0.3% hydrogen peroxide in 100% methanol for 30 minutes. After 3 washes in PBS, nonspecific reaction was blocked by incubation with PBS containing 5% skimmed milk for 30 minutes at room temperature, and then the sections were incubated with normal rabbit or goat serum for 30 min. The sections were incubated overnight at 4°C in humid chambers with the primary antibodies to CD4 and CD8 at a dilution of 1/50. After three washes with PBS, the sections were incubated with biotinylated rabbit anti-goat or rabbit immunoglobulin for 30 min. After washing again with PBS, the slides were treated with peroxidase-conjugated streptavidin for 30 min, and developed by immersion in 0.01% H_2_O_2 _and 0.05% diaminobenzidine tetrahydrochloride for 3 min. Light counterstaining with Mayer's hematoxylin was performed. The number of immunoreactive lymphocytes was counted under light microscope in a randomly selected field at the magnification of 400× in three different sections. Analysis was performed blind with respect to clinical outcome by two pathologists.

### Statistical Analysis

The associations of CR with blood cell counts and various other clinical parameters were examined using Wilcoxon's test and chi-squared test, respectively. Multivariate stepwise logistic regression analysis was performed to determine the independence of all variables identified as possibly significant. All analyses were performed with JMP8.0 software, and p-values less than 0.05 were considered to be statistically significant.

## Results

**Figure 1:**
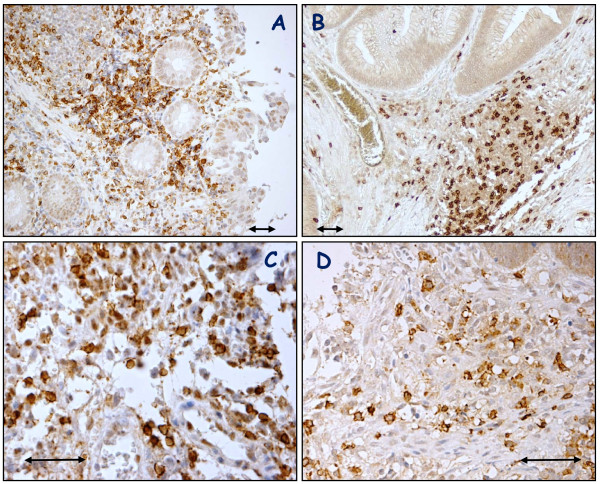
**Immunohistochemical detection of CD4(+)and CD8(+) T cells in biopsy samples of rectal cancer before CRT**. Tissue sections of biopsy samples were immunostained with anti-CD4 (A,C) or anti-CD8 (B,D) mAb. ×100 (A,B), ×400 (C,D) Arrow: 100 μm.

The number and distribution of T cells in biopsy samples before CRT were evaluated with immunostainig withm Abs against CD4 and CD8. As shown in Figure 1, both CD4 and CD8 were clearly stained in the cell membrane of interstitial infiltrates. In most cases, CD4(+) or CD8(+) T cells were evenly distributed in the whole tissue sections, while many cells clustered in specific fields in some cases. When the numbers were counted in each case, the densities of CD4(+) and CD8(+) T cells showed a strong association (data not shown).

**Figure 2:**
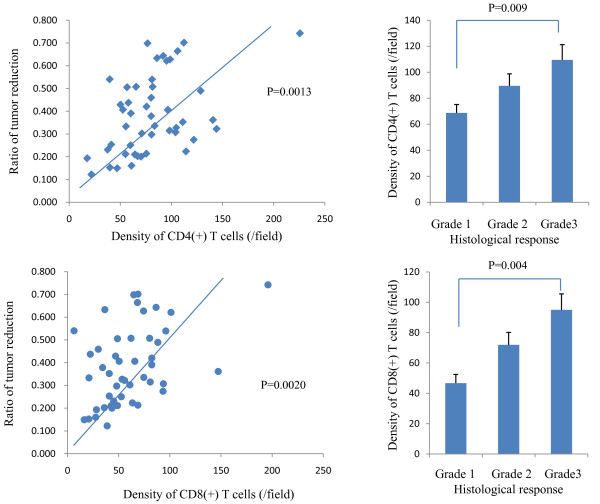
**Density of CD4(+) and CD8(+) T cells and ratio of tumor reduction and histological response to CRT.** The density of immunoreactive T cells was determined in three different biopsy samples, and mean value was calculated in each case. The longitudinal length of the rectal tumor was measured by barium enema study before and after CRT, and the ratio of tumor reduction was calculated. Histological response grade was evaluated by pathologists according to the definitions in the Japanese Classification of Colorectal Carcinoma.

More importantly, the density of CD4(+) as well as CD8(+) T cells was highly correlated with tumor response to CRT. As shown in Figure 2, the density of both CD4(+) and CD8(+) T cells showed a strong correlation with the rate of decrease of tumor size evaluated by barium enema study (P = 0.0013, 0.0020). The correlation was also observed at the histological level. As shown in Figure 2, when the cases were divided by the histological response grade according to the definitions in the Japanese Classification of Colorectal Carcinoma, the density of CD4(+) T cells was 68.7 ± 27.3/field in 27 cases of grade 1 and 89.6 ± 34.0/field in 13 cases of grade 2. Moreover, 8 cases of grade 3 contained a much higher number of CD4(+) T cells (109.5 ± 48.2/field). This trend was statistically significant (p = 0.009). Similarly, the density of CD8(+) T cells was 46.7 ± 20.7, 71.9 ± 31.9, and 95.1 ± 48.6/field in cases of grade 1, 2 and 3, respectively. (p = 0.0004).

Then, we evaluated the association between TIL density and CR. As shown in Table 1 CR cases were achieved more frequently in cases with circumferential extent less than 60% and those with size less than 4 cm on CT image. However, it was not correlated with T, N, M stage or CEA level as well as age and sex. As expected, the densities of CD4(+) and CD8(+) T cells were higher in the 8 CR cases as compared with the other non-CR cases. When the correlation was evaluated by multivariate analysis using continuous valuables, CD8(+) T cell density, but not tumor size, was an independent factor related to CR.

**Table 1:** Correlation between clinical and pathological factors and CR

Histological response		Non-CR	CR	Univariate	Multivariate
				p-value	p-value
Age		63.6 ± 10.6	60.8 ± 9.6	NS	
Sex	M	25	4	NS	
	F	15	4		
Tumor Size	≥4 cm	21	1	0.043	0.127
	<4 cm	19	7		
Circumferential tumor extent	≥60%	21	1	0.043	
	<60%	19	7		
Distance from anal verge	≥5 cm	15	5	NS	
	<5 cm	25	3		
T stage	2	12	1	NS	
	≥3	28	7		
N stage	0	26	7	NS	
	1	14	1		
M stage	0	38	8	NS	
	1	2	0		
Serum level of CEA	≥5 ng/ml	20	5	NS	
	<5 ng/ml	20	3		
CD4 density	≥78	17	7	0.015	
	<78	23	1		
CD8 density	≥54	17	7	0.015	0.0072
	<54	23	1		

NS: not significant

Size was determined by CT scan, and circumferential extent and distance from anal verge by colonoscopy. Histological grade was determined by Japanese criteria for colorectal carcinoma.

## Discussion

In this study, we found that the density of CD4(+) and CD8(+) T cells in biopsy samples of rectal cancer showed a strong correlation with tumor response to CRT, indicating that tumors attracting T cells are more liable to respond to CRT. Many previous reports have suggested that a high number of TIL in colorectal cancer is strongly associated with a favorable outcome in the patients with colorectal cancer [15-18]. Among them, the density of TIL was shown to be positively associated with response to 5-Fu chemotherapy [17]. However, in our literature search, there are no report to evaluate the correlation between TIL and radiosensitivity, and this is the first one to show the direct link between the density of T cells infiltrating in solid tumor and response to CRT.

On the other hand, Grabenbauer et al previously reported that tumor-infiltrating CD3(+) T cells, especially granzymeB(+) CD8(+) T cells, were an adverse prognostic marker for chemoradiation for anal squamaous cell carcinoma, which is totally inconsistent with our results [19]. The same negative prognostic effect of activated cytotoxic TIL accumulation was reported for Epstein-Barr (EB) virus-related nasopharyngeal tumor and Hodgkin lymphoma [20,21], which is contrary to the general findings in other tumors [16,22,23]. Since anal carcinoma is usually associated with human papilloma virus, specific viral proteins processed in tumor cells may critically affect the histological characteristics of the tumor stroma, which may account for the discrepancy between their study and ours on rectal tumors.

Although RT is widely used for the treatment of solid tumors in clinical settings, the detailed mechanisms of the antitumor effects have not been fully elucidated. Since the first report in 1979 [11], it has been proposed that tumor shrinkage is not simply dependent on direct damage to irradiated tumor cells, but is also greatly affected by the host immune response [24]. In fact, in vivo studies have suggested that cancer cells, dead or dying due to RT and/or chemotherapy, can present tumor-associated antigens to host immune cells and thereby evoke anti-tumor immune responses [25,26]. Since tumors with a higher number of tumor infiltrating T lymphocytes (TIL) are suggested to be originally immunogenic, it is speculated that CRT can further enhance the expression of so-called tumor associated antigens from those tumors, causing a better response to CRT. Our results provide further evidence suggesting a mechanical linkage between host immunity and tumor response to CRT.

The tumor response may be caused by destruction of the tumor microenvironment by CRT, which facilitates the recruitment of circulating T cells. In fact, Lugade et al have suggested that radiation-induced IFNγ production in the tumor microenvironment [27], and Matsumura el al have suggested that CXCL16 release from irradiated tumor attracts T cells [28]. In a previous study, we found that the circulating lymphocyte count is correlated with tumor response to CRT [13]. This finding is in line with the data in this study, and suggested that the maintenance of circulating lymphocytes number can recruit many anti-tumor lymphocytes into the irradiated tumor during CRT, which may lead to improvement of the clinical efficacy of RT in rectal cancer.

Taken together, our results indicate that the quantitative measurement of TIL in biopsy samples before CRT showed an independent correlation with histological as well as macroscopic tumor response to CRT, and thus can be used as a predictor of the clinical effectiveness of CRT for advanced rectal cancer. For the cases with low TIL, other anti-cancer drugs may be useful instead of 5-Fu based drugs combined with RT. Also, addition of biological response modifiers to enhance the recruitment of T cells into tumor may be critically important to improve the effectiveness of CRT.

## Competing interests

The authors declare that they have no competing interests.

## Authors' contributions

JK participated in the study design and data retrieval and analysis. KY, KK, ES participated in immunostaining and data analysis. HN participated in the management of this study. All authors read and approved the final manuscript.
